# A Compendious History of Small-Pox

**Published:** 1833-10

**Authors:** 


*A Compendious History of Small-pox; with an Account of a Mode of Local Treatment which prevents the Seaming or Scarring of the Skin, and the Occurrence of that Aggravation of Symptoms in the advanced Stages of the Disease, hitherto denominated Secondary Fever.* By HENRY GEORGE, Surgeon; Surgeon Extraordinary to H.R.H. the Duke of Gloucester. London, 1833. 8vo. pp. 112.

MR. GEORGE proposes to prevent pitting in small-pox by sprinkling calamine on the pustules, and gives four cases (which he already has had printed in the Medical Gazette,) in which he tried this plan. It succeeded in the first, third, and fourth; but, in the second, the patient died. To what unfathomable bathos medical literature may sink, we will not venture to foretell; but we have the most dismal forebodings on the subject. In the meagre work before us there is an account (at page 99) of John Grimslay's state of health on the 30th of February; and the fourth case, which is continued to the 2d of September in this year, breaks off with the words "Contin. omnia;" a conclusion of a case, and of a book, which we believe to be quite *unique*.

